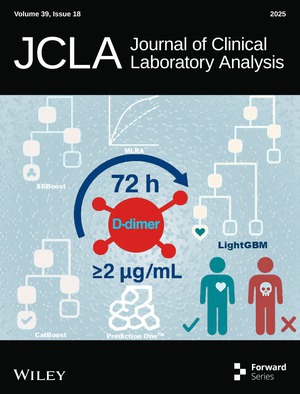# Cover Image

**DOI:** 10.1002/jcla.70112

**Published:** 2025-09-24

**Authors:** Shuma Hayashi, Ryoko Hayashi, Kayoko Nakamura, Kai Saito, Hidenori Sanayama, Takahiko Fukuchi, Tamami Watanabe, Kiyoka Omoto, Hitoshi Sugawara

## Abstract

The cover image is based on the article Routine Laboratory Tests Predict 72‐h Fatality in Patients With D‐Dimer Levels ≥ 2μg/mL: A Retrospective Cohort Study Comparing Statistical and Machine Learning Models by Hitoshi Sugawara et al., https://doi.org/10.1002/jcla.70091